# The role of crude human saliva and purified salivary MUC5B and MUC7 mucins in the inhibition of Human Immunodeficiency Virus type 1 in an inhibition assay

**DOI:** 10.1186/1743-422X-3-99

**Published:** 2006-11-24

**Authors:** Habtom H Habte, Anwar S Mall, Corena de Beer, Zoë E Lotz, Delawir Kahn

**Affiliations:** 1 Department of Surgery, University of Cape Town, Cape Town, South Africa; 2 Discipline of Medical Virology, University of Stellenbosch and National Health Laboratory Service, Cape Town, South Africa

## Abstract

**Background:**

Despite the continuous shedding of HIV infected blood into the oral cavity and the detectable presence of the AIDS virus at a high frequency, human saliva is reported to inhibit oral transmission of HIV through kissing, dental treatment, biting, and aerosolization. The purpose of this study was to purify salivary MUC5B and MUC7 mucins from crude saliva and determine their anti-HIV-1 activities.

**Methods:**

Following Sepharose CL-4B column chromatography and caesium chloride isopycnic density-gradient ultra-centrifugation, the purity and identity of the mucins was determined by SDS-PAGE and Western blotting analysis respectively. Subsequently an HIV-1 inhibition assay was carried out to determine the anti-HIV-1 activity of the crude saliva and purified salivary mucins by incubating them with subtype D HIV-1 prior to infection of the CD4^+ ^CEM SS cells.

**Results:**

Western blotting analysis confirmed that the mucin in the void volume is MUC5B and the mucin in the included volume is MUC7. The HIV inhibition assay revealed that both the crude saliva and salivary MUC5B and MUC7 mucins inhibited HIV-1 activity by 100%.

**Conclusion:**

Although the mechanism of action is not clear the carbohydrate moieties of the salivary mucins may trap or aggregate the virus and prevent host cell entry.

## Background

Several studies have shown that the human immunodeficiency virus (HIV) is not transmitted via the oral route [1]. It has also been reported that there is a continuous shedding of HIV infected blood into the oral cavity from mucosal and gingival lesions in HIV-infected patients, resulting in the detectable presence of the acquired immunodeficiency syndrome (AIDS) virus at a high frequency in the oral cavity [2-6]. However, several epidemiological studies have failed to present any conclusive evidence about oral transmission of HIV through kissing, dental treatment, biting, and aerosolization [7-14].

Extensive studies have been carried out after the initial findings of Fultz [1] that human saliva inhibited the activity of the AIDS virus [8,15,16]. These studies showed that the anti-HIV activity appeared to be highest in sub-mandibular secretions and whole saliva rather than parotid secretions [2,3,11,17-19]. Although most of these studies suggested that HIV-1 particles were aggregated by high-molecular weight components (of mucus) in both whole saliva and specifically in sub-mandibular/sublingual secretions, and which were removable by filtration through 0.45 μm pore filter [3,8,17,20,21], there has been no detailed analysis to identify the mucin components of these secretions and determine which of these components displays HIV inhibitory activity.

Thus far five secreted gel-forming (MUC2, MUC5AC, MUC5B, MUC6 MUC19), three secreted non gel-forming (MUC7, MUC8, MUC9), ten membrane bound (MUC1, MUC3A, MUC3B, MUC4, MUC11, MUC12, MUC13, MUC16, MUC17, MUC20) and three unclassified mucins (MUC14, MUC15, MUC18) have been identified [22]. Human saliva is known to contain, amongst many other factors two types of mucins, namely MUC5B and MUC7 [23], and more recently MUC19 [24]. The aim of this study was to isolate and purify these mucins in the saliva and to determine their anti-HIV-1 activity individually.

In its special report on HIV and AIDS on 30 May 2006, UNAIDS stated that since its outbreak in 1981, AIDS was found to be responsible for the deaths of more than 25 million people world-wide, with 2.8 million deaths and over 4.1 million new infections in 2005 alone [25]. This report estimated that there were more than 38.6 million HIV positive people world-wide at the end of 2005, of which two-thirds of those infected lived in Sub-Saharan Africa. Of these, South Africa with 27.9% of its adult population living with HIV [26] remains one of the worst affected countries in the world [27]. This epidemic is expected to cost South Africa 17 % of its GDP growth by 2010 [26]. With this in mind, the present study could make a significant contribution to the efforts being made in controlling this epidemic.

In this study we report the anti-HIV-1 activities of human crude saliva and purified salivary MUC5B and MUC7 mucins in an *in vitro *inhibition assay. We have shown that both crude saliva and purified MUC5B and MUC7 mucins inhibit HIV-1 activity by 100% in the range from 900 μg to 0.09 μg mucin concentration.

## Materials and methods

### Ethics

The University of Cape Town Research and Ethics Committee approved this study (ethics approval number REC REF: 283/2004).

### Materials

The ECL™ Western Blotting Detection Kit was from Amersham Biosciences (Amersham UK). Nitrocellulose membrane and dialysis tubing were from Kimix (chemical and laboratory suppliers, SA). Polyclonal rabbit anti-MUC5B (Lum 5B-2), goat anti-MUC7, goat anti-rabbit and rabbit anti-goat horse radish peroxidise (HRPO) linked secondary antibodies were from Santa Cruz Biotechnology, Inc (Santa Cruz, California). The CD4^+ ^CEM SS cells were from AIDS Research and Reference Reagent Programme (Germantown, USA). RPMI 1640, L-Glutamine and heat-inactivated fetal bovine serum were from Gibco (Massachusetts, USA). IL-2 and p24 antigen kit were from Roche Diagnostics (Germany) and Vironostika HIV-1 Antigen kit Biomérieux (France) respectively. Sepharose CL-4B and reagent solvents such as guanidinium chloride (GuHCl), phenylmethylsulfonylfluoride (PMSF), caesium chloride (CsCl), ethylenediaminetetra-acetic acid disodium salt (Na_2_-EDTA), N-ethylmaleimide (NEM), and 3-((3-cholamidopropyl)-dimethyl-ammonio)-1-propane-sulfonate (CHAPS) were from Sigma (UK). Trypan Blue Dye solution was from Merck (Germany).

### Saliva collection

Saliva was collected from healthy symptom free 'normal' female and male volunteers (who declared a risk-free lifestyle) from the laboratory who abstained from eating and drinking for at least 2 h prior to collection. Whole saliva was stimulated by chewing on parafilm and collected into 6 M GuHCl containing a spectrum of protease inhibitors (10 mM EDTA, 5 mM NEM, 1 mM PMSF) and 0.1% CHAPS pH 6.5. Samples were collected on ice and then stored at -20°C.

### Sepharose CL-4B gel filtration

Crude salivary mucus was solubilised by overnight mixing at 4°C on a revolving rotor. The insoluble debris was then separated from the soluble mucus by centrifugation at 4400 *g *for 10 min at 4°C.

Aliquots of the supernatant (20 ml) were chromatographed on a Sepharose CL-4B gel filtration column equilibrated and eluted with 4 M GuHCl containing 10 mM EDTA, 5 mM NEM and 0.05% CHAPS at pH 6.5 and at a flow rate of 48 ml/h at room temperature. Following PAS and protein (A_280_) assays, the void (V_o_) and included volume (V_i_) materials were pooled separately, dialysed against three changes of distilled water at 4°C and freeze-dried.

### Mucin preparation

For the HIV inhibition assay, crude saliva was collected into 0.1 M Tris-HCl, 2% (w/v) EDTA and 5 mM PMSF pH 7.5 (saliva:buffer ratio 1:2). After solubilisation by overnight mixing at 4°C on a revolving rotor, the insoluble debris was separated by centrifugation at 10 000 *g *for 10 min at 4°C. The supernatant was then dialysed over three changes of distilled water at 4°C and freeze-dried.

The mucin that eluted in the included volume (V_i_) of the column was subjected to caesium chloride isopycnic density-gradient ultra-centrifugation, twice for 48 h at a 105 000 g and 4°C in a Beckman L45 ultra-centrifuge [28]. Briefly, V_i _samples dissolved in 4 M GuHCl containing a cocktail of proteolytic inhibitors (as described above) were adjusted to a density of 1.39 to 1.40 g/ml with caesium chloride prior to centrifugation. Mucin rich fractions were pooled, dialysed against three changes of distilled water at 4°C and freeze-dried.

### SDS-PAGE

SDS-PAGE was carried out according to the method of Laemmli [29] in a buffer containing 0.2% SDS using 4% (w/v) stacking gels and 10% separating gels [30,31], in a Hoefer Mighty Small mini-electrophoresis system. Samples were prepared in reducing gel loading buffer containing 2% SDS, 10% glycerol, 0.01% bromophenol blue and 5% mercaptoethanol and boiled for 2 min prior to loading. After electrophoresis gels were stained for carbohydrate with PAS [32] and for protein with Coomassie Brilliant Blue G-250.

### Western blotting

After gel electrophoresis mucins were transferred to a nitrocellulose membrane (Nitrocellulose, 0.22 Micron) using a semi-dry electroblotting unit at 0.8 mA/cm^2^. The transfer buffer used contained 192 mM glycine, 25 mM Tris, 1.3 mM SDS and 20% (v/v) methanol. After electro-blotting non-specific binding was blocked by incubating the membranes overnight in 5% (m/v) low fat milk powder in TBS, 0.05% Tween-20 (TBST) at 4°C. The membranes were then washed with TBST for 3 × 5 min and incubated for 2 h with rabbit anti-MUC5B and goat anti-MUC7 polyclonal antibodies diluted in 5% (m/v) low fat milk powder in TBST at 1:500 and 1:100 dilutions respectively. The membranes were washed for 3 × 5 min with TBST and incubated for 1 h with HRPO linked secondary antibodies (goat anti-rabbit and rabbit anti-goat) diluted in 5% (m/v) low fat milk powder in TBST at 1:5000 dilution. After another TBST wash (3 × 5 min) bands that interacted with the antibodies were detected by exposing the membrane to ECL detection.

### Amino acid analysis

The amino acid contents of purified MUC5B and MUC7 mucins were analysed using a high pressure liquid chromatography (HPLC) system. The analysis procedure was similar to that of Klapper [33] and Cohen and Strydom [34]. The samples were vacuum-dried and placed in a hydrolysis vessel containing some constant boiling HCl and 1% (v/v) phenol. The vessel was cleaned with nitrogen gas and sealed under vacuum. The samples were then hydrolysed in the gas phase at 110°C for 24 h. Following hydrolysis, the vials were cooled and vacuum dried to remove the residual HCl. The dried samples were re-dissolved in citrate buffer pH 2.2 and injected into a HPLC column from Waters Associates, Medford, MA., packed with a cation exchange resin (sulfonated polystyrene cross-linked with divinylbenzene) and eluted with a series of buffers ranging from a low (0.25 M trisodium citrate, pH 3.05) to high (0.25 M sodium nitrate, pH 9.5) pH. Detection was carried out using post column derivatization with o-phthalaldehyde (OPA), a fluorescent reagent that reacts with all the amino acids except proline. The relative ratios of the individual amino acids for each sample was determined and compared to each other. For proline detection samples were treated with sodium hypochlorite prior to post column derivatization with o-phthalaldehyde (OPA).

### Toxicity assay

The toxicity of crude saliva and purified salivary MUC5B and MUC7 mucins to the CD4^+ ^CEM SS cells was determined by a toxicity assay. Briefly, 500 μl of the CD4^+ ^CEM SS cells in RPMI complete containing 10% Fetal Calf Serum, 1% Penicillin/Streptomycin antibiotic, 10 μmol Fungin and 50 μmol 2-mercaptoethanol (final concentration 2.5 × 10^6 ^cells/ml) was incubated with 250 μl of IL-2 and 250 μl (0.9 mg) of crude saliva or purified salivary MUC5B and MUC7 mucins in a CO_2 _incubator for 24 h. As controls CEM SS cells with IL-2 only and IL-2 without CEM SS cells (blank) were used. After spinning at 1400rpm for 5 min, cells were re-suspended in 500 μl of RPMI and live and dead cells were counted using Trypan blue exclusion criteria. The percentage of viable cells was calculated as live cells/total cells × 100.

### Inhibition assay

The anti-HIV-1 activities of crude saliva and purified salivary MUC5B and MUC7 mucins were tested in an inhibition assay according to the method of Nagashunmugam *et al. *[3]. Briefly, the crude saliva and purified salivary MUC5B and MUC7 mucins were dissolved in 0.25% PBS and (500 μl or 0.9 mg each) were mixed with 4 ml of the subtype D HIV-1 supernatant fluid (SNF) and incubated for 60 min at 37°C. As controls heat inactivated HIV-1 and HIV-1 plus media (RPMI 1640 with 10% fetal calf serum and IL-2) were used. The virus was first isolated from an AIDS patient by the Department of Medical Virology, Tygerberg Hospital, University of Stellenbosch, in February 1986, and it was fully characterised and sequenced subsequently. At the end of the incubation period the mixtures (virus plus mucins) and the control (virus plus media) were filtered through 0.45 μm pore size cellulose acetate filter (25 mm diameter), and both the unfiltered and filtered samples were incubated with CD4^+ ^CEM SS cells at 37°C at a concentration of 0.5 × 10^6 ^cells/ml for 30 min, 1 h and 3 h. Cells were then washed three times with PBS to remove free virus and cultured. Supernatant fluid was harvested on Day 4 and viral replication was measured by a qualitative p24 antigen assay. Endpoints were calculated by the Reed-Muench formula and the 50% tissue culture infective dose (TCID_50_) was expressed as the highest dilution that produced a positive qualitative p24 antigen result. All samples were done in triplicate and the anti-HIV-1 activity of each mucin was tested in a serial tenfold dilution (10^-1 ^to 10^-4^).

## Results

### Sepharose CL-4B gel filtration

**Figure 1:**
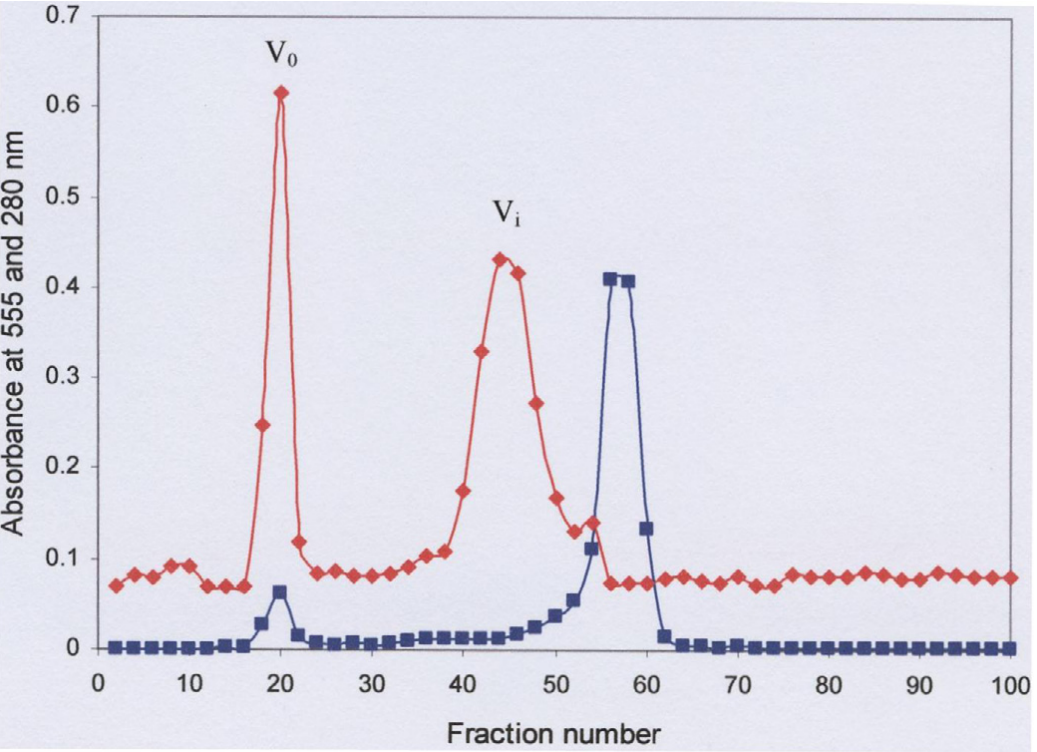
Sepharose CL-4B gel filtration of crude saliva. An aliquot (20 ml) of soluble human crude saliva extracted in 6 M GuHCl containing 10 mM EDTA, 5 mM NEM, 1 mM PMSF and 0.1% CHAPS pH 6.5 was chromatographed and eluted with 4 M GuHCl containing 10 mM EDTA, 5 mM NEM and 0.05% CHAPS pH 6.5 at flow rate of 48 ml/h at room temperature. Fractions were analysed for carbohydrate with PAS at 555 nm (◆) and for protein A_280 _(■). Materials eluted in the void volume (V_o_) and included volume (V_i_) were pooled separately, dialysed against three changes of distilled water for overnight at 4°C and freeze-dried.

Sepharose CL-4B gel filtration demonstrated that saliva contains two species of carbohydrate-rich mucin macromolecules which are distinguishable according to their size. Of these two populations, the glycoprotein (MG1) was excluded in the void volume (V_o_) [23] and the glycoprotein (MG2) in the included volume (V_i_) of the gel filtration column [23] (Fig. 1). A sharp PAS positive peak was seen eluting in the void volume and a broader peak in the included volume of the column, which was alongside a protein positive peak. The protein peak that eluted in the void volume, coinciding with the PAS positive peak, was much smaller.

### SDS-PAGE

**Figure 2:**
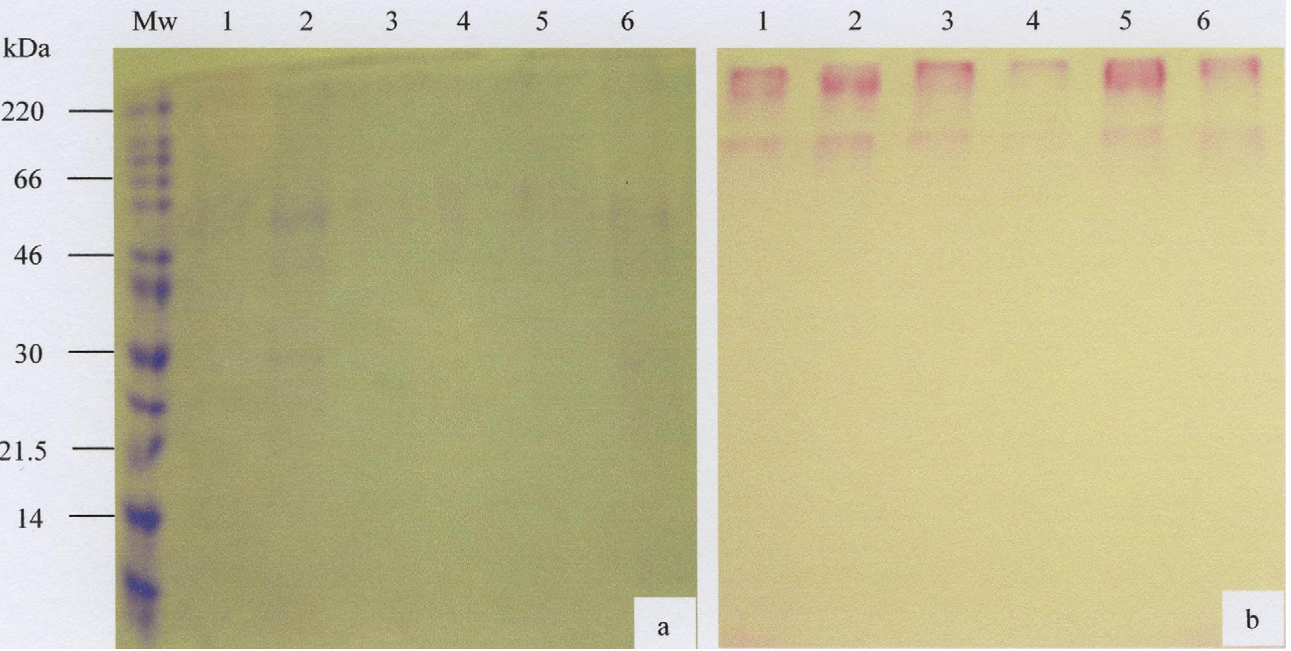
SDS-PAGE analysis of the salivary mucus eluted in the V_o _of the Sepharose CL-4B gel filtration column. Freeze-dried material (30 μg) of the V_o _prepared in reducing gel loading buffer was separated by 10% SDS-PAGE and stained with Coomassie Brilliant Blue G-250 (a) and PAS (b). The molecular weight marker is indicated by Mw and lanes 1 to 6 represents samples from 6 donors.

**Figure 3:**
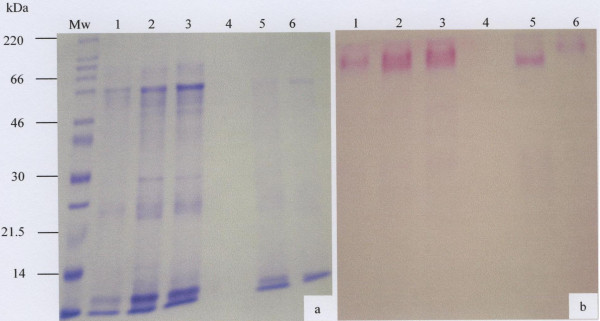
SDS-PAGE analysis of the salivary mucus eluted in the V_i _of the Sepharose CL-4B gel filtration column. Freeze-dried material (30 μg) of the V_i _prepared in reducing gel loading buffer was separated by 10% SDS-PAGE and stained with Coomassie Brilliant Blue G-250 (a) and PAS (b). The molecular weight marker is indicated by Mw and lanes 1 to 6 represents samples from 6 donors.

To check the purity of the materials eluted in the V_o _and the V_i _of the gel filtration column, fractions eluting under these peaks were pooled separately, dialysed extensively against distilled water, freeze-dried and subjected to 10% SDS-PAGE and stained for protein with Coomassie Brilliant Blue G-250 (Fig. 2a and 3a) and for carbohydrate with PAS (Fig. 2b and 3b).

The PAS positive void volume material gave a strong band sitting on the top of the stacking and running gels, very characteristic of the electrophoretic behaviour of large-sized mucins (Fig. 2b) [30]. On the other hand the material that eluted in the V_i _showed a prominent band of greater electrophoretic mobility and of relatively smaller size than V_o _mucin, in that the mucins slightly entered the running gel (Fig. 3b). Also there were a range of fainter bands ranging in size from 66–30kDa (Fig. 3b, lanes 1–3 and 5 and 6). More contaminant protein associated with mucin was present in the V_i _peak (Fig. 3a) than the V_o _peak (Fig. 2a) suggesting the need for further purification of the material that eluted in the included volume of the column.

### Mucin purification

**Figure 4:**
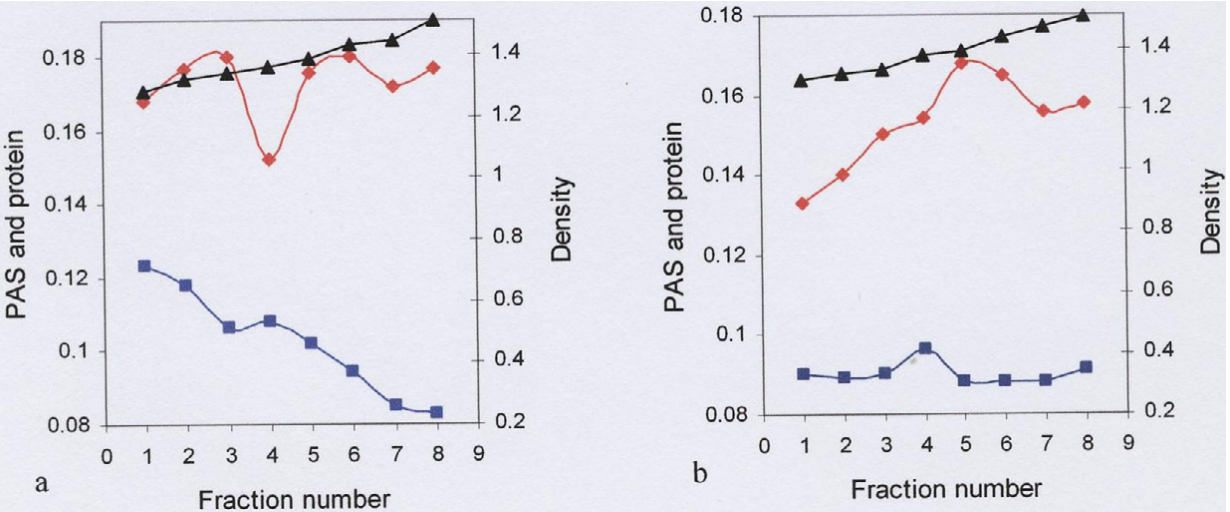
Caesium chloride isopycnic density-gradient ultra-centrifugation of the material eluted in the V_i _of the gel filtration column. Freeze-dried material eluted in the V_i _was dissolved in 4 M GuHCl containing 10 mM EDTA, 5 mM NEM and 0.05% CHAPS pH 6.5 and adjusted to a density of 1.39 to 1.40 g/ml with caesium chloride. Density gradient centrifugation was performed in a Beckman L45 ultra-centrifuge for 48 h at 105 000 g at 4°C. Fractions with high PAS (◆) and low protein (Lowry) (■) (a) were pooled and adjust to a density of 1.39 to 1.40 g/ml with caesium chloride for a second step centrifugation (b). Mucin rich fractions with a density (▲) between 1.37–1.42 were pooled, dialysed against three changes of distilled water for overnight at 4°C and freeze-dried.

**Figure 5:**
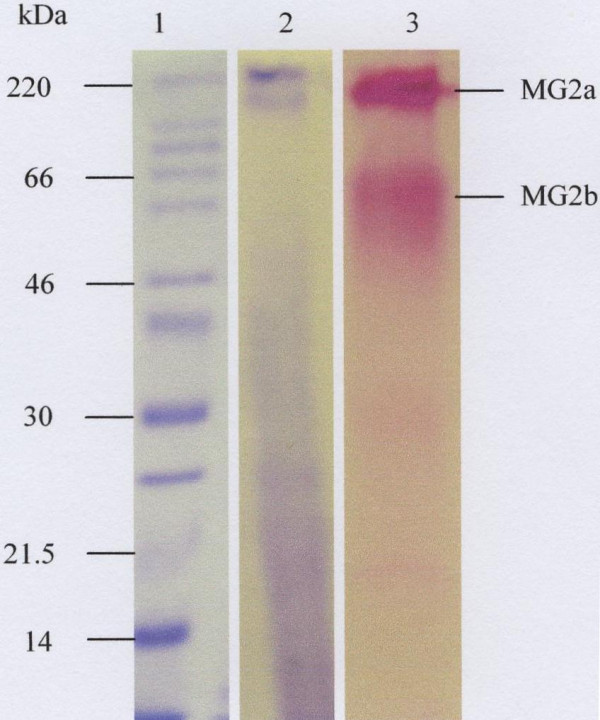
SDS-PAGE analysis of the purified V_i _material. After purification, mucins were loaded onto a 10% SDS-PAGE and stained with Coomassie Brilliant Blue G-250 (lane, 1 and 2) and PAS (lane, 3). Molecular weight markers and the two mucin isoforms are indicated by lane 1and the arrows respectively.

Material that eluted in the included volume of the gel filtration column was subjected to density-gradient ultra-centrifugation in caesium chloride. Mucin rich fractions from the first density gradient (Fig. 4a) were pooled and subjected to a second spin in caesium chloride (Fig. 4b), after which the mucin rich fractions were pooled, dialysed against distilled water and freeze-dried. SDS-PAGE analysis of these mucins showed less protein contaminant (Fig. 5, lane 2), except for low molecular weight material of 30kDa and under. As indicated by the arrows, the PAS stain of the same material gave two prominent bands in the region of 220kDa and 46–60kDa (Fig. 5, lane 3). Traces of material were also seen at 21.5kDa and under (Fig. 5, lane 3).

### Western blotting

**Figure 6:**
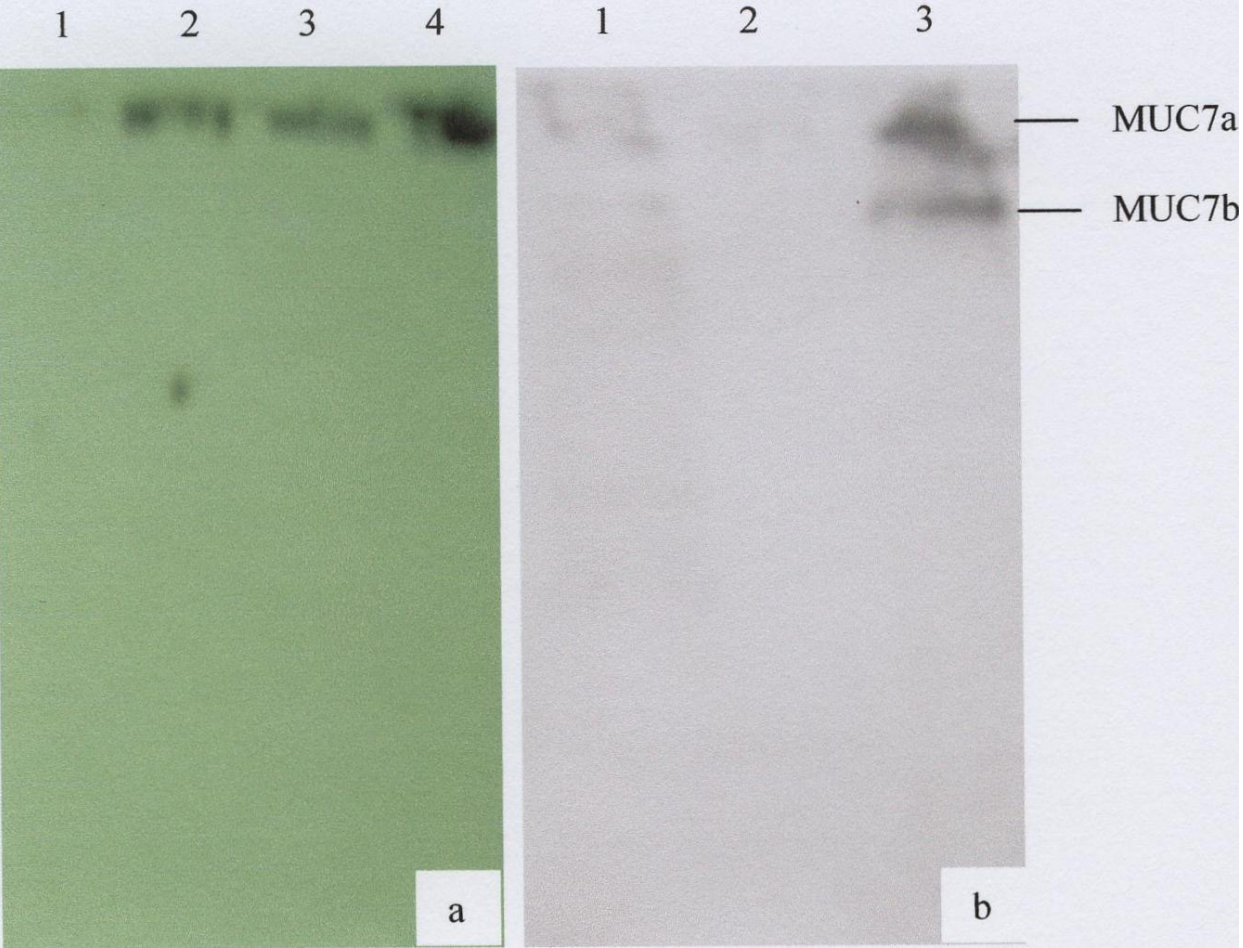
Western blotting analysis of Vo and Vi material from the gel filtration column using rabbit anti-MUC5B and goat anti-MUC7 polyclonal antibodies. a) Lanes 1, MUC7 (negative control), 2 and 3, V_o _material from two donors and 4, respiratory MUC5B (positive control) and in b) Lanes 1, crude saliva (positive control), 2, gastric mucin (negative control) and 3, purified V_i _material were separated by 10% SDS-PAGE and transferred to nitrocellulose membrane. Following overnight blocking, the membranes were incubated for 2 h with rabbit anti-MUC5B (a) and goat anti-MUC7 (b) polyclonal antibodies. Membranes were then incubated for 1 h with goat anti-rabbit (a) and rabbit anti-goat (b) HRPO linked secondary antibodies and bands that interacted with the antibodies were detected by exposing the membrane to ECL detection. The arrows indicate the two glycoforms of MUC7.

To determine the identity of the V_o _and V_i _mucins from the gel filtration column material eluting under these peaks was subjected to 10% SDS-PAGE and then transferred to nitrocellulose membrane and probed with rabbit anti-MUC5B and goat anti-MUC7 polyclonal antibodies respectively. The results showed that the V_o _material sitting on the top of the gel is MUC5B (Fig. 6a, lanes 2 and 3) and the two bands slightly entered the running gel that interacts with the anti-MUC7 polyclonal antibody are the two glycoforms of MUC7 (Fig. 6b, lane 3). The two glycoforms of MUC7 in Figure 6b are indicated by arrows.

### Amino acid analysis

Following the amino acid analysis (Table 1) both MUC5B and MUC7 mucins were found to contain higher amounts of threonine, serine, glutamic acid, glycine and aspartic acid. However, the amount of threonine in MUC7 is very low. Of these signature amino acids of mucins, serine, threonine, and proline were found to comprise 26% and 28% of the MUC5B and MUC7 mucins respectively.

**Table 1:** Amino acid composition of MUC5B and MUC7 mucins.

Amino acids	MUC5B (mole %)	MUC7 (mole %)
Aspartic acid	8.9	9.1
Threonine	10.0	4.0
Serine	7.4	5.5
Glutamic acid	9.0	12.4
Proline	8.5	18.6
Glycine	9.0	14.4
Alanine	6.6	3.9
Valine	6.4	4.2
Methionine	1.6	1.7
Isoleucine	4.0	2.9
Leucine	7.6	4.7
Tyrosine	3.2	1.7
Phenylalanine	3.8	1.9
Lysine	4.6	4.7
Histidine	4.4	3.9
Arginine	4.0	5.6

### Toxicity assay

Prior to the HIV inhibition assay the toxicity of the crude saliva and purified salivary MUC5B and MUC7 mucins to the CD4^+ ^CEM SS cells was determined by toxicity assay. Based on this assay (data not shown) no cell death or toxicity of these mucins to the CD4^+ ^CEM SS cells was detected.

### Inhibition assay

**Figure 7:**
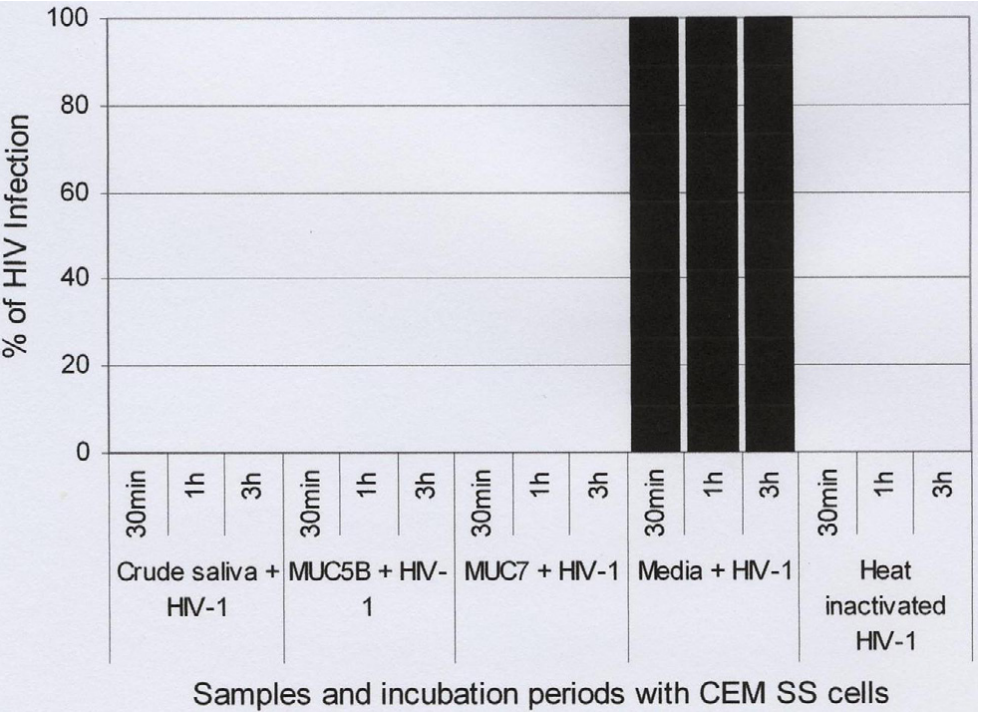
Inhibition of HIV-1 activity by human crude saliva and purified salivary MUC5B and MUC7 mucins. Crude saliva and purified salivary MUC5B and MUC7 mucins (0.9 mg each) were incubated with subtype D HIV-1 for 60 min and filtered through 0.45 μm pore size cellulose acetate filter. As controls HIV-1 treated with media and heat inactivated HIV-1 were used. The unfiltered samples were then incubated with CD4^+ ^CEM SS cells at a concentration of 0.5 × 10^6^cells/ml for 30 min, 1 h and 3 h. After PBS wash cells were cultured and viral replication was measured by a qualitative p24 antigen assay.

Subsequent to the toxicity assay the anti-HIV-1 activities of the crude saliva and purified salivary MUC5B and MUC7 mucins were determined by HIV inhibition assay. The result revealed that after a 30 min incubation period during which the mixtures three separate mixtures (HIV-1 plus crude saliva), (HIV-1 plus MUC5B) and (HIV-1 plus MUC7) were incubated with the CD4^+ ^CEM SS cells, no HIV-1 infection of these cells was observed (Fig. 7). Simultaneously, to determine the effect of the incubation period between the CD4^+ ^CEM SS cells and the mixtures described above on the infectivity of these cells, incubation was carried out at different time-points (1 h and 3 h). No difference in the rate of inhibition due to time differences was detected (Fig. 7). Serial tenfold dilution (10^-1 ^to 10^-4^) of the mucins was also done to determine the anti-HIV-1 activity of these mucins at the highest dilution. All the mucins showed anti-HIV-1 activity down to the concentration of 0.09 μg or in the range of 900 μg to 0.09 μg (data not shown).

In the controls, where the virus was incubated with the media only, prior to addition to the CD4^+ ^CEM SS cells at all time points (30 min, 1 h and 3 h), 100% of HIV-1 replication or infection of the CD4^+ ^CEM SS cells was detected (Fig. 7). However no HIV-1 infection was revealed when heat inactivated HIV-1 was used (Fig. 7).

**Figure 8:**
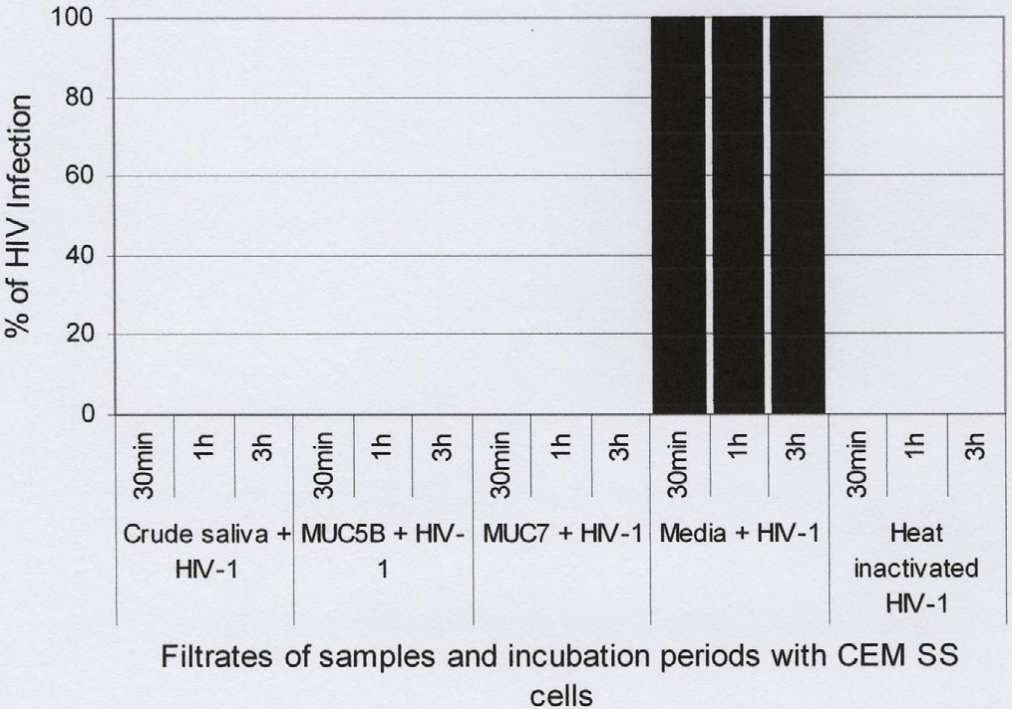
Inhibition of HIV-1 activity by human crude saliva and purified salivary MUC5B and MUC7 mucins. Crude saliva and purified salivary MUC5B and MUC7 mucins (0.9 mg each) were incubated with subtype D HIV-1 for 60 min and filtered through 0.45 μpore size cellulose acetate filter. As controls HIV-1 treated with media and heat inactivated HIV-1 were used. The filtrates of the mixtures were then incubated with CD4^+ ^CEM SS cells at a concentration of 0.5 × 10^6^cells/ml for 30min, 1h and 3h. After PBS wash cells were cultured and viral replication was measured by a qualitative p24 antigen assay.

To determine whether the HIV-1 activity of the crude saliva and purified salivary MUC5B and MUC7 mucins is by aggregation or trapping of the virus, the mixtures (HIV-1 plus crude saliva), (HIV-1 plus purified salivary MUC5B), (HIV-1 plus purified salivary MUC7) and (HIV-1 plus media), at the end of the 60 min incubation period, were filtered through 0.45 μm pore size cellulose acetate filter (25 mm diameter) and the filtrates were incubated with the CD4^+ ^CEM SS cells at all time-points (30 min, 1 h and 3 h). While 100% of viral replication or infection of the CD4^+ ^CEM SS cells was detected with the filtrate of the control (HIV-1 plus media) (Fig. 8), no HIV-1 replication or infection of the CD4^+ ^CEM SS cells was detected when the filtrates from the three mixtures were used (Fig. 8).

## Discussion

In the present study human crude saliva was separated by Sepharose CL-4B gel filtration column into material eluting as two major PAS positive peaks in the void (MG1) and included (MG2) volumes respectively and the material under each peak identified as MUC5B and MUC7 respectively, confirming previous findings of Thornton *et al *[23]. Amino acid analysis further supported this material to be mucin [35]. The small protein peak that eluted in the void volume of the gel filtration column, under the large excluded PAS positive peak is very likely the protein moiety of MUC5B (Fig. 1) [31]. The elution of the larger protein peak, which begins under the included volume (V_i_) PAS positive peak and elutes nearer the total volume of the column suggests that gel filtration was partially but not entirely successful in removing a considerable amount of protein contaminant from the crude saliva. SDS-PAGE analysis of the PAS positive V_i _peak material, when stained for protein with Coommassie Blue, still showed protein contamination of MUC7 (Fig. 2c). This material was therefore further purified by caesium chloride density gradient ultra-centrifugation.

To avoid any possible endogenous enzymatic degradation, sample analysis was carried out in 6 M GuHCl containing protease inhibitors such as EDTA, NEM, and PMSF. According to the report by Carlstedt *et al. *[36] while PMSF and EDTA inhibit serine and metallo-proteases respectively, NEM which inhibits thiol proteases is reported to reduce disulfide bond formation between mucins and non-mucinous components of the saliva by blocking the free thiol groups on mucins.

Salivary mucins are known to bind with the non-mucinous components of the saliva such as amylase, lysozyme, proline-rich protiens, statherin, histatins, SIgA and lactoferrin which may change their physicochemical properties [37-39]. As reported by Mehrotra *et al. *[40] and Zalewska *et al. *[41] the hydrophobic regions of mucins are responsible for these complex formations as well as for their sticky characters. As a result mucins may stick to gels and dialysis tubes during the purification and dialysis processes. Therefore in the present study CHAPS a detergent was included in the chromatography buffer to minimize the sticky characteristic and prevent loss of mucins during dialysis and column chromatography [42].

The electrophoretic behaviour of the purified saliva was typical of mucins which, because of their large size, appear at the top of the stacking and running gels (Fig. 2b and 3b, and Fig 5, lane 3) [31,43,44]. The MUC7 mucin showed two bands on SDS-PAGE (Fig 5, lane 3) and Western Blotting (Fig. 6b, lane 3). This result agreed with that of Reddy *et al. *[45], Mehrotra *et al. *[40] and Bolscher *et al. *[42], which revealed the presence of two isoforms of MUC7, designated MUC7a and MUC7b in saliva. According to these authors the two isoforms have an identical amino acid composition but a different glycosylation pattern with respect to sialic acid and fucose. In the case of MUC5B, although SDS-PAGE analysis revealed the presence of two closely situated mucin bands on the top of the gel (Fig. 2b), Western blotting gave just one band (Fig. 6a, lanes 2 and 3), again at the top of the membrane.

The amino acid analysis has shown the presence of higher amounts of threonine, serine, glycine, glutamic acid, and aspartic acid in salivary MUC5B and MUC7 mucins. This agreed with the findings of Tabak *et al. *[46] that, mucins contain large amounts of serine, threonine, proline, glutamic acid, glycine and alanine. The presence of methionine in a very small amount also agreed with that of Thomsson *et al. *[47]. Strangely enough threonine presented in smaller amount than the expected value especially in MUC7. According to Mehrotra *et al. *[40], this could be due to the level of oligosaccharide substitution of this residue.

It has been shown that the incubation of HIV with human whole and submandibular saliva leads to a decrease in viral infectivity and that this inhibition is considerably reduced upon filtration of the saliva [11,15,18]. These observations suggest that saliva-induced viral aggregation serves to clear the virus from the oral cavity and lessen the possibility of oral transmission [11]. It has also been suggested that the most likely candidate in the oral defence against AIDS is a macromolecular component [9], specifically the mucus component of oral secretions [2]. Although these researchers speculated that crude saliva [8], MUC5B and MUC7 [2], two of the known mucins in saliva [23] are potentially involved in inhibition through the entrapment of virus particles, a definitive attempt to purify, identify and individually test these purified mucins from saliva, against the AIDS virus, has not been done.

Although the HIV-1 Subtype C virus is currently the most prevalent in South Africa, the Subtype D which was used in this study was found during the early HIV epidemic in South Africa and is still prevalent in the country, although less frequently. Although we would very much have liked to use the Subtype C strain, unfortunately, the Subtype D is the only laboratory adapted strain available for an *in vitro *HIV assay, in the entire country. As described in the methods, this virus was first isolated from an AIDS patient by the Department of Medical Virology, Tygerberg Hospital (Cape Town, South Africa) in February 1986, and fully characterised and sequenced subsequently. Simultaneously the T cell lines, CEM-SS cells which were used in this experiment are known to produce distinct and repeatable syncytia formation when infected with HIV-1. As reported by Nara *et al. *[48], following the addition of HIV-1, these cells develop easily quantifiable syncytia formation in four to six days.

In this study we have demonstrated that when the HIV-1 was incubated with the crude saliva and purified salivary MUC5B and MUC7 mucins and subsequently added to the CD4^+ ^CEM SS cells no viral replication or infection of the CEM SS cells was detected by the p24 antigen assay. However, when the HIV-1 was incubated with media instead (as control), 100% infection of the CD4^+ ^CEM SS cells was detected. A possible explanation of the findings of this study is that when mucins are incubated with HIV, the mucins trap or aggregate the virus, leaving it entangled in the mucin mesh which is retarded by filtration through a 0.45 μm pore cellulose acetate filter [3,8,17,20,21], thus preventing the virus from entering the host cells.

To prove this hypothesis we filtered the mixtures (HIV-1 plus crude saliva), (HIV-1 plus MUC5B), (HIV-1 plus MUC7) and the control (HIV-1 plus media), at the end of the incubation period, through a 0.45 μm pore size cellulose acetate filter, and added the filtrates to the CD4^+ ^CEM SS cells. The filtrates from each of the mixtures failed to infect the CD4^+ ^CEM SS cells, as shown by the absence of measurable p24 activity. This suggested that the mucins aggregated all the viruses, hence leaving no free viruses in the filtrates to cause viral infection. However, the filtrates from the control (HIV-1 plus media) where mucins are absent, gave 100% HIV-1 replication or infection of the CEM SS cells. Therefore the media, unlike the mucins failed to aggregate the viruses, resulting in free viruses in the filtrate, which were then infective to the CD4^+ ^CEM SS cells.

Although the inhibition of HIV-1 by mucins is thought to be by aggregation of the virus prior to host cell entry [3,8,11,18], the specificity of this aggregation is not at all clear. The findings by Fox *et al. *[9] and Nagashunmugam *et al. *[3], which demonstrated low or no potency of saliva against herpes simplex virus (HSV), HIV-2, simian immunodeficiency virus, Epstein-Barr virus, cytomegalovirus, hepatitis B virus and adenovirus implies that the specific aggregation of HIV-1 by saliva or purified mucins could require some specificity. One other possibility is that the negative charges on mucins through sialic acid and sulphate groups [35] could be responsible for specific interactions with receptors on the virus.

This study has shown that mucus and mucins have protective properties against the HI virus in certain situations. More light needs to be shed on these processes to understand further the role of mucins against infection to be able to design further studies that will investigate the significance of mucins in HIV-AIDS.

## Authors' contributions

HH carried out the biochemical studies and drafted the manuscript. AM conceived of the study, participated in its design and coordination and finalised the manuscript. CdB established and carried out the HIV inhibition assay. ZL participated in the biochemical studies. DK contributed ideas to the design and coordination of the study. All authors read and approved the final manuscript.
